# Seatbelt syndrome associated with an isolated rectal injury: case report

**DOI:** 10.1186/1749-7922-5-4

**Published:** 2010-02-04

**Authors:** Ashraf F Hefny, Yousef I Al-Ashaal, Ahmed M Bani-Hashem, Fikri M Abu-Zidan

**Affiliations:** 1 Department of Surgery, Al-Ain Hospital, Al-Ain, PO Box 1006, UAE; 2 Department of Surgery, Faculty of Medicine and Health Sciences, United Arab Emirates University, Al-Ain, PO Box 17666, UAE

## Abstract

Seatbelt syndrome is defined as a seatbelt sign associated with a lumbar spine fracture and a bowel perforation. An isolated rectal perforation due to seatbelt syndrome is extremely rare. There is only one case reported in the Danish literature and non in the English literature. A 48-year old front seat restrained passenger was involved in a head-on collision. He had lower abdominal pain and back pain. Seatbelt mark was seen across the lower abdomen. Initial trauma CT scan was normal except for a burst fracture of L5 vertebra which was operated on by internal fixation on the same day. The patient continued to have abdominal pain. A repeated abdominal CT scan on the third day has shown free intraperitoneal air. Laparotomy has revealed a perforation of the proximal part of the rectum below the recto sigmoid junction. Hartmann's procedure was performed. The abdomen was left open. Gradual closure of the abdominal fascia over a period of two weeks was performed. Postoperatively, the patient had temporary urinary retention due to quada equina injury which resolved 10 months after surgery. The presence of a seatbelt sign and a lumbar fracture should raise the possibility of a bowel injury.

## Background

Despite the decreasing mortality in restrained victims of motor vehicle collisions (MVC), a new type of injury related to seatbelt usage has emerged. Seatbelt sign is the linear ecchymosis of the skin caused by the seatbelt following MVC [1]. Seatbelt syndrome is defined as a seatbelt sign associated with a lumbar spine fracture and a bowel perforation. An isolated rectal perforation due to seatbelt syndrome is extremely rare. There is only one case reported in the Danish literature and non in the English literature [2].

## Case presentation

**Figure 1:**
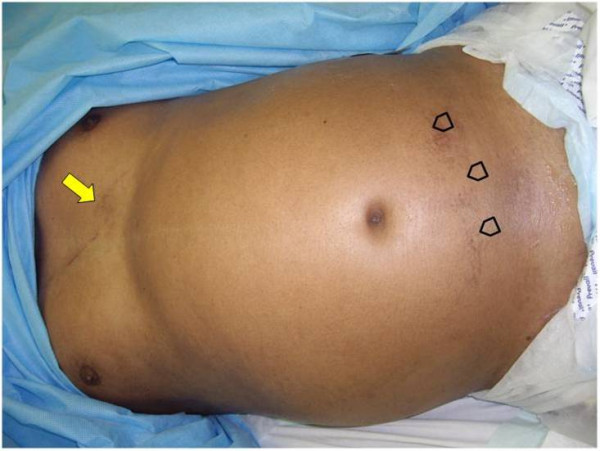
**Seat belt sign crossing obliquely through the chest (arrow) and transversely through the lower abdomen (arrow heads)**.

**Figure 2:**
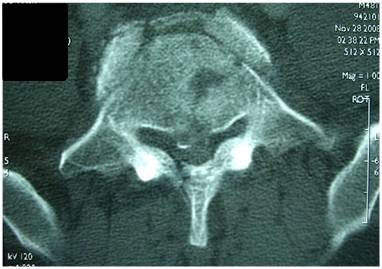
**Burst spine fracture of L5**. There was narrowing of more than 60% of the spinal canal, three column fracture involving the body and right lamina with posterior bulging of a bone fragment into the canal.

**Figure 3:**
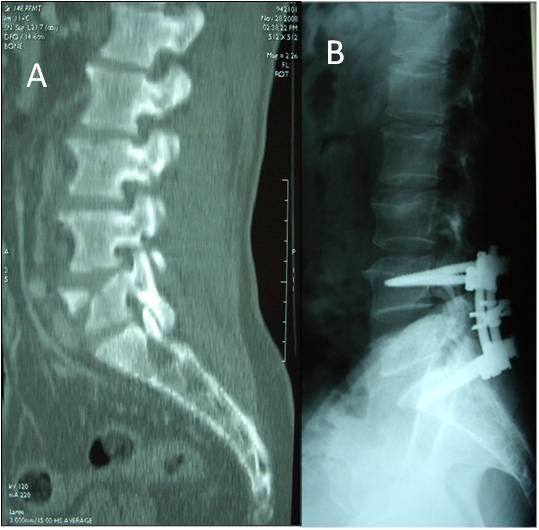
**Sagittal reconstruction of the lumbosacral spine (A) showing the burst fracture of L5 (A)**. This was internally fixed using a pedicle screw instrumentation through a posterior approach to achieve extension and distraction (B).

**Figure 4:**
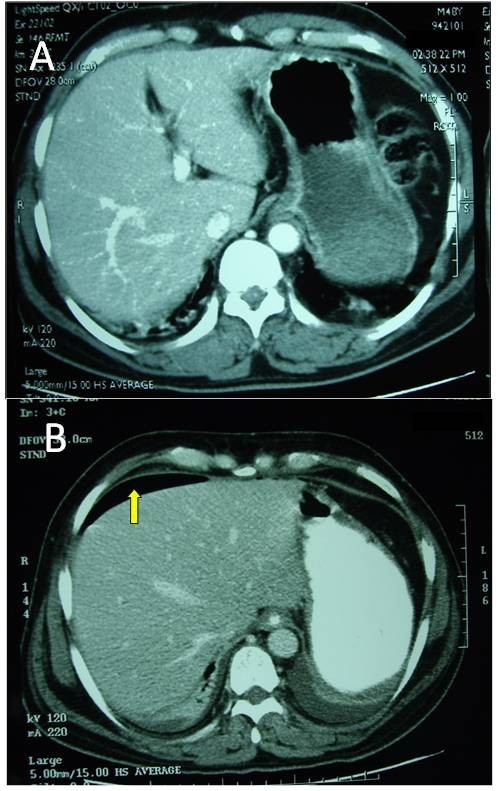
**Abdominal CT scan with intravenous contrast on day 1 (A) which was normal and on day 3 (B) which showed free intraperitoneal air (arrow) and left pleural effusion**.

**Figure 5:**
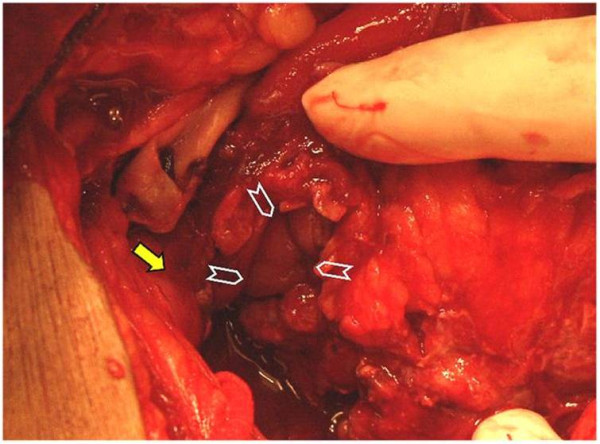
**Rectal perforation at the rectosigmoid junction (arrow heads)**. The perforation was below the pelvic rim (arrow).

A 48-year old front seat restrained passenger was involved in a head-on collision. He has presented with lower abdominal pain and back pain. Seatbelt mark was seen transversely across the lower abdomen (Fig 1). There was partial weakness of the muscle power of the right lower limb. Initial trauma CT scan was normal except for a burst fracture of L5 vertebra. There was narrowing of more than 60% of the spinal canal, three columns fracture involving the body and right lamina with posterior bulging of a bone fragment into the canal (Fig 2). This fracture was internally fixed using a pedicle screw instrumentation and a laminectomy on the same day of admission through a posterior approach to achieve extension and distraction (Fig 3). The patient continued to have abdominal pain and distention which became evident on the third day. Bedside ultrasound has shown distended small bowel loops without evidence of intraperitoneal fluid. Repeated abdominal CT scan with intravenous contrast has shown free intraperitoneal air. Furthemore, there was distended thickened small bowel loops. There was a low attenuation area anterior to the left psoas muscle suggesting of inflammatory changes but no free intraperitoneal fluid could be demonstrated. There was bilateral pleural effusion more on the left side (Fig 4). Exploratory laparotomy has revealed the presence of free intrapeitoneal air but there was no faecal soiling. The small bowel was hugely distended, thickened and inflamed. A perforation of the proximal part of the rectum which was below the recto sigmoid junction was covered by small bowel loops (Fig 5). Hartmann's procedure was performed with end colostomy. Huge distention of the bowel loops made it impossible to close the abdomen. The abdomen was left open and temporarily closed using saline IV bags sandwiched between two layers of Steri-Drape. The patient was taken to the operating theatre four times over a period of two weeks where the abdominal cavity was gradually closed. Postoperatively, the patient had urinary retention due to quada equina injury but he could walk. The patient travelled back into his home country where he had closure of the colostomy and reinstalling the continuity of the colon. Follow up after 10 months of the injury showed that the patient was walking and controlling both his urination and daefecation.

## Discussion

Injury of the colon and rectum following blunt trauma is rare and its early diagnosis is difficult [3]. Restrained patients of MVCs with seatbelt sign have more incidence of intestinal injury than others [4]. Intestinal injury should be strongly suspected in patients with a seatbelt sign associated with a lumbar fracture (seat belt syndrome) [5,6]. Computed tomography (CT) has shown to be the diagnostic test of choice for the evaluation of blunt abdominal trauma in haemodynamically stable patients [7]. Finding bloody stool or blood per rectal examination mandates proctosygmoidscopy [3]. Some rectal injuries can be detected after contrast enema [8].

There is no reliable diagnostic test that can completely exclude intestinal injury in blunt abdominal trauma when immediately done after trauma [9]. In equivocal abdominal examinations, diagnostic peritoneal lavage may help in detecting intestinal perforation, but similarly, it may also miss the injury if it was performed soon after trauma [7]. Clinical suspicion and serial physical examinations are essential in detecting such injuries. The presence of an associated lumbar vertebral fracture makes the clinical abdominal assessment difficult and unreliable [10]. Repeated CT scan after 8 hours in suspected cases may help in early diagnosis of bowel perforation [7]. In our patient, the abdominal CT scan was repeated due to persistent abdominal pain and distension. It has shown free intraperitoneal air. At laparotomy, perforation of the proximal part of the rectum was detected. This is a very rare seatbelt complication [2]. It is difficult to explain how the rupture occurred under the pelvic rim although there was no pelvic fracture in this patient. This injury was not iatrogenic by the pedicle screws as the screws did not penetrate beyond the bodies of the vertebrae as shown by figure 3. Furthermore, the rectal perforation was only in the anterior wall of the rectum while the posterior wall was intact. Pedicle screw internal fixation was indicated because the patient presented with a neurological deficit, unstable fracture and narrowing of the spinal canal of more than 50% [11-13]

The only way we could explain the mechanism of this rectal injury is by sudden increase of the intra luminal pressure of a closed bowel loop by the seatbelt during deceleration. This can result in a bursting injury with perforation [7,14]. The same mechanism has been proposed for oseopahgeal rupture caused by a seatbelt injury [14]. A distended closed bowel loop is especially susceptible to rupture when its wall is stretched because of the tri-axial stress effect. In contrast, if it was empty, a larger force is required to cause its rupture [15,16].

In cases of delayed diagnosis of large bowel perforation, Hartmann's procedure is safer and more effective [17]. Delayed diagnosis of intestinal perforation increases the incidence of sepsis and its associated morbidity and mortality [10,18]. Primary closure of the abdominal fascia is ideal but it was impossible in our patient. The development of abdominal compartment syndrome was a real concern because of the distension and oedema of the inflamed bowel. The abdomen was left open and gradually closed [19]. The technique we have used is cheap, controls fluid and heat loss, does not adhere to the abdominal wall and simplifies re-exploration of the abdomen with decreased mortality [20]. Despite that, the abdominal domain may be lost as the edges may retract with a risk of evisceration if the abdominal wall closure was delayed [19,20].

## Conclusions

The presence of a seatbelt sign and a lumbar fracture should raise the suspicion of a bowel injury. Seatbelt injury can cause rectal perforation. Repeated serial clinical examination is essential to avoid missed bowel perforations.

## Consent

Written informed consent was obtained from the patient for the publication of this case report. A copy of the written consent is available for review by the Editor-in-Chief of this journal.

## Competing interests

The authors declare that they have no competing interests.

## Authors' contributions

AH assisted in the operation and follow-up of the patient, collected the literature, wrote the manuscript and approved the final version of the manuscript. YA helped in the idea, operation, follow-up of the patient, data collection and approved the final version of the manuscript. AB helped in the idea, data collection and writing of the manuscript, and finally, FA performed the repeated abdominal surgery, had the idea, and assured the quality of data collected, helped draft the first version of the paper, repeatedly edited it, and approved the final version. All authors read and approved the final manuscript.
